# Risk assessment for commercial crime and legal protection based on modularity-optimized LM and SCA-optimized CNN

**DOI:** 10.7717/peerj-cs.1683

**Published:** 2023-11-16

**Authors:** YunZhe Wang, FengLan Su

**Affiliations:** HeBei Sport University, Shijiazhuang, Hebei, China

**Keywords:** Commercial crime, LM, CNN, SCA, Legal protection

## Abstract

With the Internet of Things (IoT) making significant strides in recent years, the challenges associated with data collection and analysis have emerged as a pressing concern in public security. When employed to tackle extensive criminal networks, the conventional deep learning model encounters issues such as heightened computational complexity, sluggish operational efficiency, and even system failures. Consequently, this research article introduces an intricately devised framework for detecting commercial offenses, employing a modularity-optimized Louvain-Method (LM) algorithm. Additionally, a convolutional neural networks (CNN)-based model is formulated to determine the feasibility of extending legal aid, wherein feature transformation is facilitated by utilizing TFIDF and Word2vec algorithms aligned with diverse legal text corpora. Furthermore, the hyper-parameter optimization is accomplished using the sine cosine algorithm (SCA), ultimately enabling the classification of relevant legal guidance. The experimental outcomes comprehensively affirm the exceptional training effectiveness of this model. The commercial crime identification model, grounded in modular optimization as proposed in this article, adeptly discerns criminal syndicates within the commercial trading network, achieving an accuracy rate exceeding 90%. This empowers the identification of such syndicates and bestows the judicial sphere with pertinent legal insights.

## Introduction

In recent years, with the continuous development of the economy and society, the situation of comprehensive social governance has become increasingly serious. Commercial crime is becoming increasingly prominent, and it is easy to induce major social risks. Leaders at all levels of the state attach great importance to this. General Secretary Xi Jinping made important instructions to crack down on various crimes, stressing the need to strengthen key rectification and strictly curb the spread trend of various crime problems. To improve the people’s sense of security and life happiness index, the public security departments continue to promote the governance of various criminal problems and always maintain a high pressure on various criminal activities. Through the strong investigation and control of public security units at all levels, remarkable results have been achieved in the fight against various crimes (Feng, 2020). To maximize data’s leading and early warning role in comprehensive social governance, we should gather massive amounts of information, such as people flow, capital flow, information flow, and call flow, and conduct deep mining to form relevant forecasts (Stevenson & Slobogin, 2018). At the same time, public security organs are facing the challenge of explosive data growth, especially with the increasingly mature development of the Internet of things in recent years, leading to more serious problems of data collection, analysis and processing faced by public security. Faced with such problems and challenges, public security organs are often passive in solving public security data analysis and prediction. How to fully excavate valuable information from public security data and effectively predict criminal behavior is the key to improving the public security system’s work efficiency and reducing the risk to public security.

Compared with human decision-making, the automatic decision-making algorithm has relatively objective, fair and efficient characteristics, so its application has gradually spread to all fields of social life (Deng et al., 2021; Goel et al., 2021; Cai et al., 2021). For example, a university in China uses the algorithm to identify students’ economic status according to consumption records to help determine the payment of subsidies for poor students; Banks widely use algorithms to evaluate customers’ credit to decide whether to make loans; The US Department of Education uses algorithms to determine whether the teacher employment contract is renewed; In the courts of the United States, some of the crime risk assessment algorithms are used by judges. The automatic decision-making of algorithms directly affects the basic rights of human beings in social security, medical care, supervision of public officials and judicial system.

The traditional algorithm faces a significant dilemma when it comes to regulating paths primarily because it tends to overlook the contextual aspects of the algorithm itself. This oversight is a critical issue, as the behavior and performance of an algorithm can vary significantly depending on the specific context in which it operates. Various factors, such as the subject matter it is applied to, the nature of the objects it interacts with, and the unique problems it seeks to address, all play a pivotal role in shaping the algorithm’s characteristics and outcomes.

The challenges and complexities have become increasingly pronounced in our ever-evolving landscape of comprehensive social governance. Society now grapples with multifaceted issues spanning various sectors, from public policy and law enforcement to economic and commercial activities. These challenges are further compounded by the growing prominence of commercial crimes, which have become more sophisticated and widespread.

In light of these pressing concerns, it becomes evident that a one-size-fits-all approach to algorithm regulation is no longer sufficient. Regulatory frameworks must adapt to acknowledge the nuanced interplay between algorithms, subjects, objects, and problems to effectively address the intricate and dynamic issues of our modern world. Only by doing so can we hope to harness the potential benefits of algorithms while mitigating their potential risks and ensuring that they serve the best interests of society as a whole.

This article formulates a legal decision-making model leveraging Convolutional Neural Networks (CNN). The model employs the Tf-idf and Word2Vec algorithms to transform features from various legal text corpora and fine-tune hyperparameters using the SCA to classify pertinent legal guidance, reducing the risk to public security.

## Related Works

Algorithm prediction relies on the existing feature data and feature information. It establishes a data model for these data through a classification or clustering algorithm and calculates and predicts according to the model. Kshatri et al. (2021) proposed that association rules can be used to find potential association rules from criminal behavior information or criminal records to analyze and predict crimes and also to analyze the relationship between different types of cases and the characteristics of cases, as well as the relationship between different types and criminal methods. Although the crime prediction is successfully realized, there are still some defects. For example, the algorithm based on association rules is FP-growth, which will cost a lot of memory and is not widely available. In addition, the K-Means algorithm is sensitive to the initial centroid, and improper selection of the initial centroid will easily lead to the phenomenon that the convergence centroid process is very long and the execution time of the algorithm is too long. Safat, Asghar & Gillani (2021) advocated the utilization of Support Vector Machines (SVM) for crime prediction. They posited the viewpoint that SVM tackles the solution process as a convex quadratic programming problem, thereby capable of providing global solutions. This makes SVM a viable choice for crime prediction.

However, the voluminous data required for crime prediction presents a substantial challenge. The SVM algorithm, when confronted with a sizable training dataset, demands substantial memory and extends the algorithm’s execution time considerably. In addition, the classical support vector machine algorithm only furnishes two classification methods, rendering it less adept at tackling multi-classification problems. Neil and his colleagues posit that the current escalation in crime rates necessitates a departure from traditional, sluggish crime resolution techniques. Consequently, they advocate the deployment of machine learning and computer vision algorithms to aid law enforcement in forecasting crimes, alleviating the police force’s burden and contributing to crime prevention (Neil, Nandish & Manan, 2021). It is worth noting, however, that while they suggest the utility of machine learning and computer vision algorithms for crime prevention, they have yet to implement this proposal.

On the other hand, Shen et al. (2019) proposed utilizing the naive Bayesian algorithm to assess and compute the conditional probabilities of each feature attribute amenable to classification. The data set is then categorized based on these attribute-specific conditional probabilities, thereby achieving the classification and crime prediction objective. Nevertheless, it is imperative to recognize that the naive Bayes algorithm assumes attribute independence, a presumption that does not always hold in practical application and real-life scenarios. Furthermore, Lan did not undertake optimizations to enhance the naive Bayes algorithm. Suppose a particular attribute value remains absent in the training set. In that case, its prior probability value defaults to zero, which can cause the naive Bayes calculation formula to yield zero, impacting the final classification outcome and diminishing the Bayesian algorithm’s accuracy. Lastly, Pu et al. (2023) advocated using a spatiotemporal coarse-grained algorithm for crime prediction. Her approach entails extracting diverse time components through time series decomposition, sequential input of these components into the most suitable algorithms, and, ultimately, their effective integration.

To achieve high-performance training data, the GPU used is relatively high-end and expensive, and it also needs to be equipped with better CPU, SSD and RAM. It can be seen that deep learning requires very high machine configuration. However, the Hadoop cluster runs on some cheap commercial hardware.

Hence, executing deep learning algorithms on Hadoop clusters, particularly those with modest configurations, exerts significant computational stress on the hardware. Conversely, machine learning operates proficiently with a standard CPU for effective data training, demonstrating no particular hardware demands. Within this study, we employ a scanning algorithm to scrutinize crime patterns and assess their characteristics. Nonetheless, establishing the parameters for the DBSCAN algorithm poses a challenging task. Inadequate parameter selection can affect clustering outcomes, especially in scenarios with numerous data dimensions.

Furthermore, when confronted with high data dimensionality, DBSCAN exhibits suboptimal performance. Pan has proposed using the KNN algorithm to construct a predictive model for crime classification  (Zhongying, 2019). However, the KNN algorithm entails extensive computations and exhibits limited fault tolerance. In cases where sample data for a particular class is scarce, it can lead to reduced classification accuracy for those specific classes. Kounadi conducted a comprehensive analysis of 32 papers from 2000 to 2018, culminating in synthesizing and appraising cutting-edge spatial crime prediction technologies (Kounadi, 2020). Gaurav introduced a spatiotemporal crime prediction technology rooted in machine learning and two-dimensional hotspot analysis. They assert that each crime category correlates with time, weather, location, and demographic parameters (Hajela, Chawla & Rasool, 2020).

## Commercial Crime Identification Model based on Modularity-Optimized LM

Commercial crime cannot be separated from money transactions, so the capital transaction relationship between accounts in a commercial network can be effectively characterized by calculating transaction weight. Suppose the calculated capital transaction weights are applied to the Louvain-Method (LM) algorithm. The suspected subnets with the same fund transaction characteristics and close fund transactions can be effectively divided in that case. LM is a powerful community detection algorithm used in network analysis to identify clusters of nodes with strong internal connections. This algorithm maximizes modularity, a measure of network partition quality. LM starts with an initial partition and then iteratively optimizes modularity by moving nodes between communities. It aggregates the network into a new representation, repeating the process until convergence. Finally, it assigns nodes to communities in the aggregated network. LM’s efficiency and scalability make it ideal for uncovering hidden structures in complex networks across various domains, from social networks to biology and transportation planning.

The LM algorithm can be considered a clustering algorithm to partition a large-scale network into distinct communities. Nodes within a community exhibit similar characteristics and maintain strong connections, whereas nodes bridging different communities exhibit distinct characteristics and sparse connections (Tian et al., 2022). Due to its good classification accuracy and excellent adaptability to large-scale networks, the LM algorithm is slowly applied to the scene of commercial network crime identification based on capital transactions (Bahulkar et al., 2018). However, the original LM algorithm is serial. When applied in large-scale financial networks, it is easy to lead to slow operation efficiency and even system crashes. To solve this problem, we need to optimize the modularity of the original LM algorithm.

### Modularity optimization process

As an important index to measure the reasonable degree of community division, the main idea is in a random business network, the probability of capital transaction relationship between two accounts (that is, there are edges between two nodes) is equal. It has the same number of nodes as the actual network, but the edges in the random network are connected randomly. By comparing the proportion of the number of edges of each community node with the total number of edges in the actual network, subtracting the expected difference of the total number of edges in the community of the corresponding random network. If the difference is larger, the randomness is smaller, the relationship between nodes in the network is closer, and the situation of community division is better (Seifikar, Farzi & Barati, 2020). Therefore, we can define modularity as follows:

(1) \begin{eqnarray*}Q= \frac{1}{2m} \sum _{i,j}\, \left\vert {A}_{ij}- \frac{{k}_{i}{k}_{j}}{2m} \right\vert \delta \left( {c}_{i},{c}_{j} \right) \end{eqnarray*}

where m represents the number of edges in the network, *A*_*ij*_ represents the weight of edges between node I and node j, indicating the closeness between node i and node j. *k*_*i*_ is the sum of the values of all edges connected to node i, and $ \frac{{k}_{i}{k}_{j}}{2m} $ represents the closeness of i and j when the network is random. *c*_*i*_ is the cluster number of node i. If *c*_*j*_ is the same, then $\delta \left( {c}_{i},{c}_{j} \right) =1$, indicating that node i and node j are in the same cluster. Otherwise, $\delta \left( {c}_{i},{c}_{j} \right) =0$, indicating that node i and node j are not in the same cluster. $ \frac{1}{2m} $ is the introduced inertia factor, which is to control the value of modularity within the interval (0,1).

According to the idea of modularity, the definition of community modularity gain is given as follows:

(2) \begin{eqnarray*}\delta Q= \left[ \frac{\sum in+{k}_{i,in}}{2m} -{ \left( \frac{\sum tot+{k}_{i}}{2m} \right) }^{2} \right] - \left[ \frac{\sum in}{2m} -{ \left( \frac{\sum tot}{2m} \right) }^{2}-{ \left( \frac{{k}_{i}}{2m} \right) }^{2} \right] \end{eqnarray*}

The above formula can be simplified as

(3) \begin{eqnarray*}\delta {Q}^{{}^{{^{\prime}}}}={k}_{i,in}- \frac{\sum tot\times {k}_{i}}{m} \end{eqnarray*}

whereas, *k*_*i*,*in*_ signifies the summation of weights about cluster C linked to node i, and ∑ tot symbolizes the cumulative sum of total weights connected to cluster C. Additionally, *k*_*i*_ denotes the cumulative sum of total weights linked to node i. It is noteworthy that the simplified calculation yields a relative gain rather than an absolute gain. This transformation significantly mitigates programming complexity.

**Figure 1:**
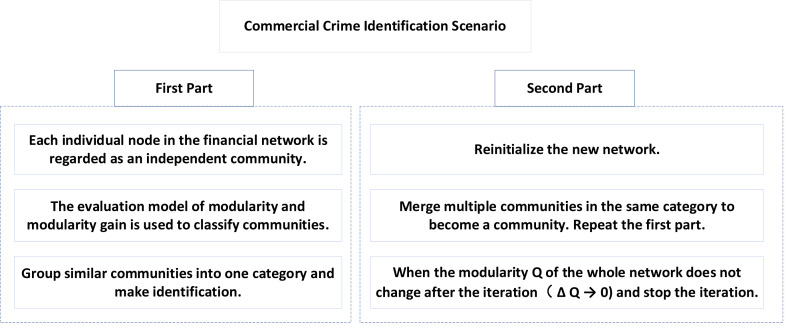
Commercial crime identification scene.

As shown in Fig. 1, the application of the LM algorithm based on modularity in the scene of commercial crime identification is divided into two parts.

In the first part, each node in the financial network is regarded as an independent community, and these communities are classified by the above evaluation model of modularity and modularity gain, and similar communities are classified into one group and identified.

In the second part, we reinitialize the new network, merge multiple communities into a community, and then repeat the first part to complete a round of iteration. When the modularity Q of the whole network no longer changes after iteration (*δQ* → 0), the iteration stops.

### Implementation process

**Figure 2:**
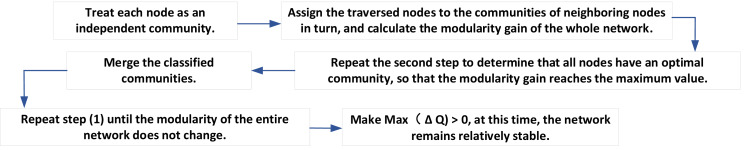
Algorithm steps.

The specific steps of the above algorithm are shown in Fig. 2.

 (1) Each node in the transaction network is regarded as an independent community.

(2) After traversing each node of the transaction network, the nodes traversed are allocated to the community of adjacent nodes in turn, and the modularity gain of the whole network *δQ* is calculated if max(*δQ*) > 0, that is, there is an adjacent node of the current node. After merging the current node into a community, the module degree of the whole network increases, while if it does not exist max(*δQ*) > 0. The current node does not join the community where any node is located, and the original state remains unchanged (Zhang et al., 2021).

(3) Repeat Step (2) to determine that all nodes in the network have an optimal community so that the network modularity gain reaches the maximum value.

(4) In this way, all the nodes belonging to the same community are merged into one node. The weights of the edges between the nodes in the community are transformed into the weights of the new nodes, and the weights of the edges between the communities are transformed into the weights of the edges between the new communities.

(5) Repeat Step (1) until the modularity of the whole network is no longer changed; that is, there is no merging of any two communities so that max(*δQ*) > 0. At this time, the network remains relatively stable.

In traversing the intricate web of transaction nodes within the network, a systematic procedure is followed to determine the allocation of these nodes to their respective adjacent communities (Niu et al., 2023). This allocation step plays a pivotal role in assessing the community structure’s robustness and optimizing it for enhanced modularity, a key measure of network organization. At each juncture of node traversal, the algorithm assesses the potential benefit of reallocating the current node to a different community. This evaluation hinges on the concept of “modularity gain”, which quantifies the improvement in the network’s modularity when a node is moved to a new community. In this context, modularity measures how densely connected the nodes are within a community compared to what would be expected by chance.

In the implementation of the community discovery algorithm, the most complex and time-consuming task is the community division in step (2) because there are a large number of nodes participating in the calculation, and the modularity gain of the whole network needs to be calculated when the current node joins each neighbor node. In principle, the community division of each node can be optimized with the idea of parallelization because the modularity change is only related to the community of the current node and the neighbor node of the current node and has nothing to do with other communities. When step (2) is executed, the order of traversing nodes has little influence on the final community clustering effect (Linhares et al., 2020).

## Design of Legal Assistant Decision Model

### Overall design

**Figure 3:**
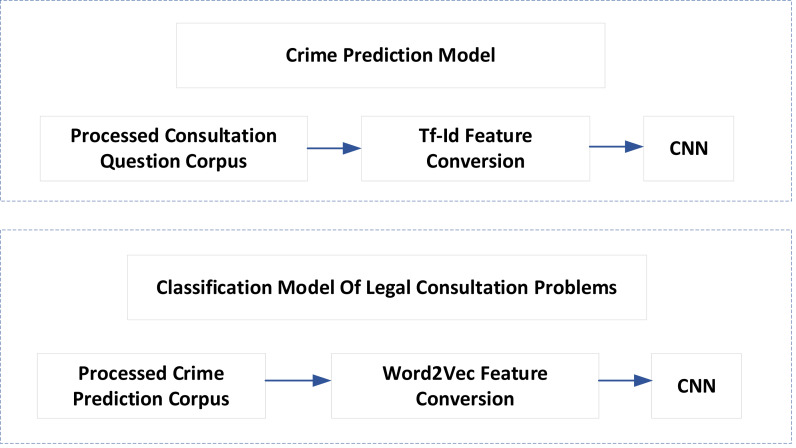
Legal assistant decision model.

Artificial intelligence technology can meet the needs of many judicial aspects, such as intelligent conviction, classification of legal advisory issues, document recommendation, *etc*. The task of intelligent conviction is to use the computer to read the case description and automatically give the appropriate charges. The classification task of legal consultation is to predict which legal level the problem may belong to based on the description of the user problem. The task of document recommendation is to search for similar documents from massive data to assist judges and lawyers in judging cases. Given the identification of commercial crimes, this article proposes a corresponding legal assistant decision-making model, as shown in Fig. 3. The model mainly solves two problems: one is crime prediction, and the other is the classification of legal advisory issues.

### CNN algorithm design

#### Input layer

In the input layer of the convolutional neural network, to realize the representation from single word feature to whole document feature, a more intuitive feature organization method is adopted. For a document, a combination of several words is formed after processing. After each word is transformed into a word vector, and then each word vector is arranged vertically, the text data can be converted into matrix data.

After the text is processed, suppose that a text contains m words, and a corresponding word vector represents each word; then, the text can be expressed as a vertical arrangement of m f-dimension vectors, and f is the dimension set by the word vector during the training process. Afterword segmentation and stop word filtering, the longest legal text in the training set contains 935 words. The word embedding length of each word is set to 300 dimensions, that is, m = 935, k = 300. Therefore, the corresponding input layer organization is a 935 * 300 network structure. Text data often has no fixed length; some may be shorter. 935 is just the longest text length in all data sets. The text that cannot reach the length needs to be processed separately. In this article, the method of zero padding is used. For a text with n words (*n* < 935), the first n words are arranged vertically by word embedding, and the insufficient parts are filled with zeros, *i.e.,* 935-n zeros.

#### Convolution layer

The input of the convolution layer is a text matrix; each row of the matrix represents a word, and the matrix size is 935×300 (Lin et al., 2020). Convolution kernel extracts features by sliding on the text matrix. When the width of the convolution kernel remains unchanged, the height of the convolution kernel represents the size of the convolution kernel. If the text matrix is defined as *A* ∈ *R*^*s*∗*d*^ and the size of the convolution kernel is h, then the weight vector of the convolution kernel is *w* ∈ *R*^*h*∗*d*^, then there are *h*^∗^d parameters to be calculated. Line i to line i + h − 1 of the text matrix can be expressed as A[i:i + h − 1]. When the convolution window acts on the text submatrix A[i:i + h − 1], the following formula can be obtained:

(4) \begin{eqnarray*}{\mathrm{o}}_{\mathrm{i}}=\omega \cdot \mathrm{A}[\mathrm{i}:\mathrm{i}+\mathrm{h}-1].\end{eqnarray*}

Among them, *i* = 1…, *s* − h + 1, the symbol ⋅ represents the dot product operation. If the convolution kernel operates on the entire sentence matrix, then the length of the output *O* is *s* − *h* + 1. Then, the deviation term *b* ∈ *R* and the activation function *f* are used to work on each. *O*_*i*_, then *C*_*i*_ can be obtained. The semantic feature map *c* ∈ ℝ^*s*−*h*+1^ can be obtained by all *C*_*i*_. The calculation formula of *C*_*i*_ is as follows:

(5) \begin{eqnarray*}{c}_{i}=f \left( {O}_{i}+b \right) .\end{eqnarray*}

In order to improve the training speed, The Relu function is used as the activation function in this article:

(6) \begin{eqnarray*}f(x)=max(0,x).\end{eqnarray*}

#### Full connection layer

The full connection layer is usually at the tail of the convolutional neural network, and all neurons have a weight connection. According to the K features with the greatest influence, the feature data is divided into a one-dimensional vector and sent to the full connection layer with the Softmax classifier (Ma, He & Peng, 2023) so that all the local features extracted can be considered comprehensively to complete the classification task of the model.

### Super parameter optimization

In this article, SCA is proposed to optimize the parameters, that is, to find the most suitable set of parameters in the range of optional parameters so that the model’s accuracy is the highest. In the operation of SCA, it is necessary to determine the fitness function. In this experiment, the accuracy of the model on the test set is selected as the fitness function, as shown in Eq. (7)

(7) \begin{eqnarray*}\mathrm{acc}(f;D)= \frac{1}{m} \sum _{\mathrm{i}=1}^{\mathrm{m}}\,\mathrm{II} \left( \mathrm{f} \left( {\mathrm{x}}_{\mathrm{ i}} \right) ={\mathrm{y}}_{\mathrm{i}} \right) \end{eqnarray*}

To balance the exploration and development phases and the range of sine and cosine in the equation. The following equation is used for adaptive changes:

(8) \begin{eqnarray*}{\mathrm{r}}_{1}=\mathrm{a}-\mathrm{t} \frac{\mathrm{a}}{\mathrm{T}} \end{eqnarray*}

where t is the current iteration, T is the maximum number of iterations, and a is a constant. The specific optimization steps are as follows:

(1) The processed and transformed data are divided into a test set and a training set, which are used for model training and testing. The hyper-parameter range of the prediction model was determined.

(2) Initialize SCA and set the parameters of SCA.

(3) Execute the first iteration of SCA, where the fitness value is calculated according to the fitness function, and the solution with the highest fitness in the current initial solution set is selected and saved.

(4) When t is 2, the parameter r1 is calculated and the position of the candidate solution is updated.

(5) After the new candidate solutions are obtained, the fitness of each candidate solution is calculated. The optimal fitness in this iteration is selected for comparison with the candidate solutions saved in the last round. If the current candidate solution is superior to the previous one, the current candidate solution is selected. Otherwise, the solution from the previous iteration is selected.

(6) Judge whether the termination conditions are met, and if so, proceed to the next step; Otherwise, make *t* = *t* + 1 and repeat Step (5) and Step (6).

(7) When the optimal fitness solution and the location of the solution are obtained, the hyper-parameters are output, and the parameters are input into the model for training to get the final model.

## Experiment and Analysis

### Experimental data

In this research endeavor, the experimentation process relied on authentic bank production data, underscoring the validity and practicality of the findings. The experiment was conducted meticulously and precisely, leveraging a genuine data set sourced from a banking institution. This data set, accessible at the following link (https://doi.org/10.7272/Q61G0JHJ), comprises a comprehensive and extensive collection of financial records.

To provide a snapshot of the data’s magnitude, it encompasses a staggering 78,760 distinct accounts, embodying a diverse cross-section of financial activity. Within the confines of a single month, an astonishing 568,978 transaction records were meticulously logged and analyzed. Each transaction record is replete with a rich tapestry of information, containing 70 distinct fields, each offering valuable insights into the intricate world of financial transactions.

It is imperative to note that the bank, in a conscientious effort to safeguard customer privacy and protect sensitive financial information, has taken stringent measures to ensure data security. Consequently, the real production data set has been transformed into a secure and confidential format. This transformation includes desensitization, which involves removing or obfuscating personally identifiable information, and encryption, which fortifies the data against unauthorized access. These measures collectively ensure that the integrity and confidentiality of the data are upheld, complying with the highest standards of data protection and privacy regulations.

By employing this authentic and rigorously prepared data set, the study aimed to provide a robust foundation for its analyses, insights, and conclusions. The reliance on actual production data enhances the relevance and applicability of the research findings, ultimately contributing to a deeper understanding of the intricate dynamics surrounding commercial network criminal gangs and their activities within the realm of capital transactions.

### Model training effect

**Figure 4:**
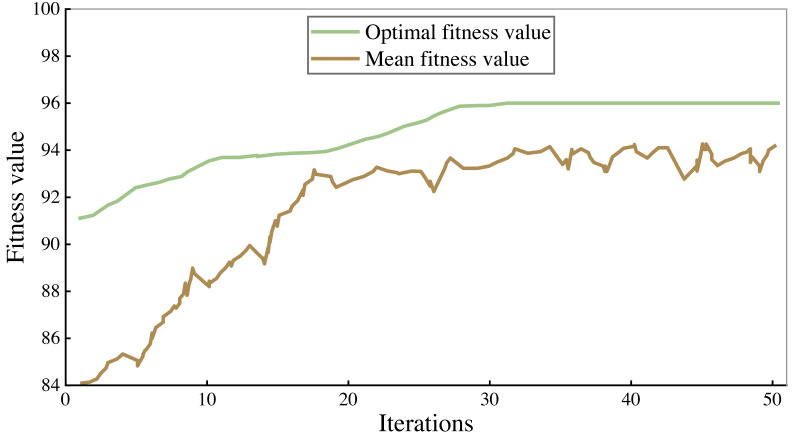
Training results of commercial crime identification model.

**Figure 5:**
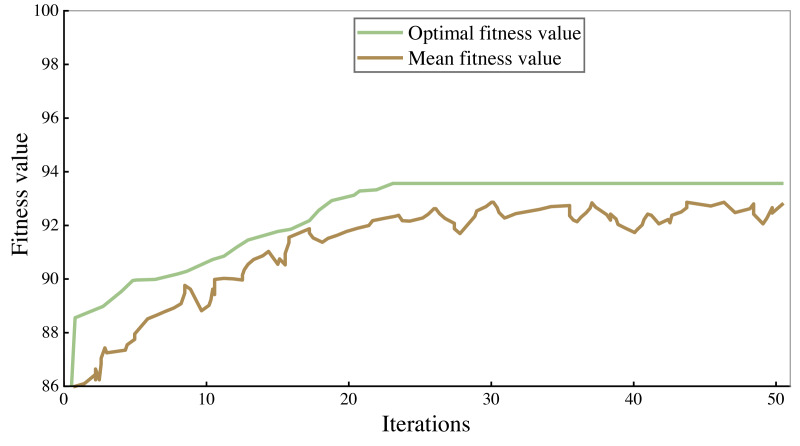
Training results of legal assistant decision model.

All the corpus was divided into 7:3 parts, 70% of which were used to train the model and 30% to verify the performance of the model. The fitness curves in the model training process are shown in Figs. 4 and 5, and accuracy is used to evaluate them.

As can be seen from the figure, the accuracy rate of the legal assistant decision model is maintained at 94%–95%, and that of the commercial crime identification model is maintained at 92%–93%. As the number of iterations increases, the fitness value will also increase. When the number of iterations reaches 25–30, the fitness value tends to be stable. After 50 iterations, the algorithm stops. Finally, the highest accuracy rate of the legal aid decision model is 95.93% and 93.66% of the commercial crime identification model.

### Comparison of different models

**Figure 6:**
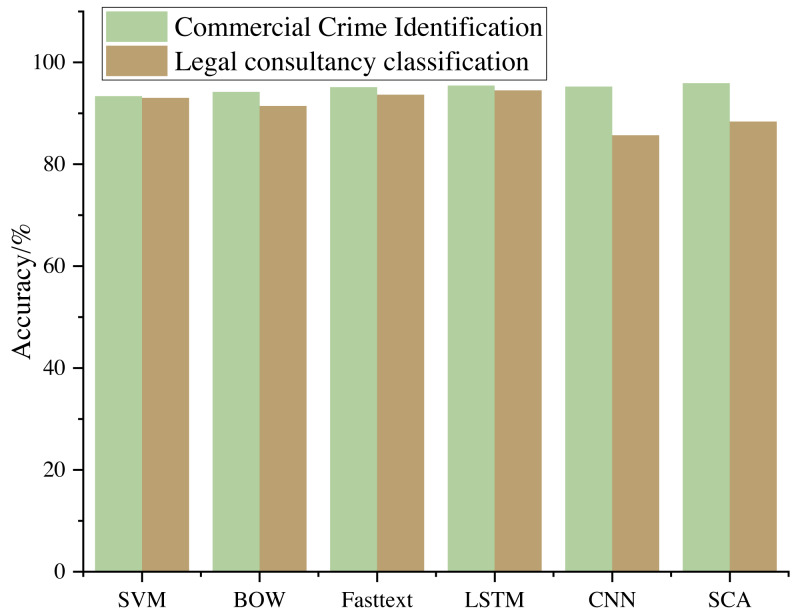
Model comparison results.

The commonly used algorithm models are selected for comparison, and the comparison results are shown in Fig. 6.

The research outcomes unequivocally demonstrate the superiority of deep learning methodologies over traditional machine learning algorithms in commercial crime identification. This empirical evidence underscores the potential of advanced neural network-based techniques in tackling complex real-world challenges. Specifically, the deep learning approach exhibited remarkable performance gains compared to its traditional counterparts.

Notably, within the realm of deep learning, a significant enhancement in accuracy was observed with the utilization of an optimized CNN as opposed to a CNN with manually configured hyperparameters. This optimization process underscores the importance of fine-tuning neural network architectures to suit the specific task. By carefully tuning the hyperparameters, the algorithm achieved higher accuracy, further underlining the capacity for deep learning to adapt and excel in intricate problem domains.

Conversely, a stark contrast emerged when comparing deep learning, represented by CNN, with the traditional random forest algorithm. While the deep learning approach exhibited substantial advantages in terms of accuracy, it is essential to acknowledge that deep learning algorithms may not always yield ideal results in all scenarios. One key reason for this disparity is the nature of the legal consultation questions. These questions tend to be concise and lack substantial linguistic connections. Consequently, the deep learning algorithms face the challenge of discerning and classifying these short sentences, often relying on just a handful of crucial keywords.

In summary, these findings emphasize the evolving landscape of machine learning, where deep learning techniques are proving their mettle in specialized tasks like commercial crime identification. However, the data’s intricacies and the problem’s specific characteristics must be considered when selecting the appropriate algorithm. The unique challenge posed by succinct legal consultation questions highlights the importance of continuous refinement and adaptation in achieving optimal algorithmic performance.

### Commercial crime identification effect

**Figure 7:**
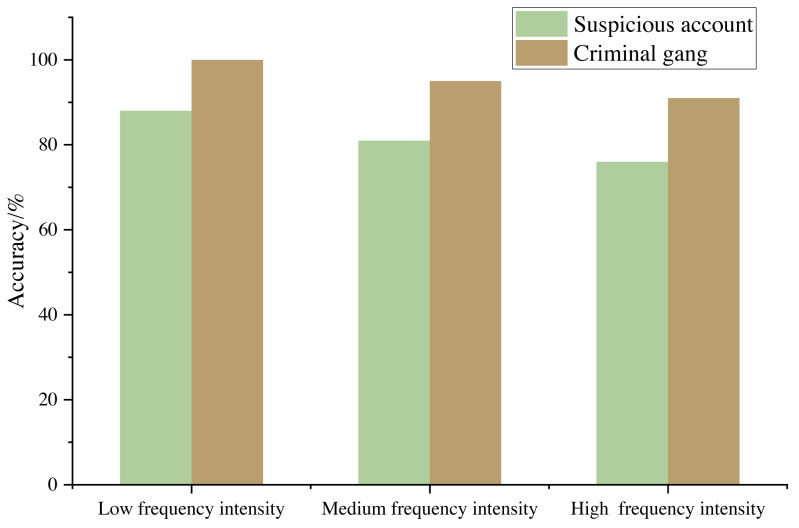
Commercial crime identification effect under different sample frequency intensity.

According to the suspicious report and the later investigation certificate, the commercial criminal account provided by the bank is taken as the real criminal account. The suspicious account in the scope of the real criminal account obtained by the algorithm in this article is regarded as the experimental suspicious account, and the account not within the scope of the real criminal account is regarded as the experimental false alarm account. In the commercial transaction network, the Adjusted Rand Index (ARI) is used to measure the accuracy of the results. The experimental results are shown in Fig. 7.

Figure 7 shows that the commercial crime identification model based on modular optimization proposed in this article can identify the criminal gangs in the commercial trading network, and the accuracy rate is more than 90%. When the fund transaction between accounts is characterized by low amount frequency, the model performs better because the intimacy weight between accounts becomes smaller and the number of suspicious accounts decreases, which indicates that the method meets the expectation and the experimental results are reasonable.

## Conclusion

This study develops a legal assistant decision-making model based on CNN and a commercial crime identification model based on modular optimization, realizing the precise identification of criminal gangs in the commercial transaction network and through legal informatization. The test data in this research use genuine bank production data sets to complete the experiment. The findings demonstrate that the model has excellent accuracy in identifying commercial crime and classifying legal consultation problems, which can assist managers in analyzing commercial security data and enhancing the productivity of the legal profession. This study has successfully laid the foundation for advanced applications in legal assistance and commercial crime identification. Moving forward, several promising avenues for further research and development can build upon these achievements.

Firstly, the legal assistant decision-making model based on CNNs shows great potential for broader legal informatization initiatives. Future investigations could delve into expanding the scope of this model to encompass a wider array of legal tasks and domains. This could involve fine-tuning the model to handle more complex legal scenarios, such as contract analysis, legal document classification, or predictive legal decision-making.

Similarly, the commercial crime identification model based on modular optimization presents an exciting opportunity for refinement and expansion. Further research could explore the application of this model in diverse contexts beyond commercial crime, such as fraud detection in financial systems, network security, or even cybersecurity. Additionally, the optimization techniques used in this model could be adapted to improve the efficiency and effectiveness of other machine learning algorithms, potentially leading to advancements in various domains.

Moreover, using genuine bank production data sets in this research opens avenues for developing more comprehensive and nuanced datasets tailored to legal and financial domains. Creating such datasets with carefully curated and labeled examples could facilitate training even more accurate and specialized models.

## Supplemental Information

10.7717/peerj-cs.1683/supp-1 Supplemental Information 1 Code

## Additional Information and Declarations

### Data Availability

The following information was supplied regarding data availability: The code is available in the Supplementary File. The dataset is available at Dryad: - Rotering, Thomas; Apollonio, Dorie (2022). Cannabis industry businesses, organizations, and individuals that lobby in Colorado [Dataset]. Dryad. https://doi.org/10.7272/Q61G0JHJ.
